# Neutral colonisations drive high beta-diversity in cavernicole springtails (Collembola)

**DOI:** 10.1371/journal.pone.0189638

**Published:** 2018-01-02

**Authors:** Cristina Fiera, Jan Christian Habel, Werner Ulrich

**Affiliations:** 1 Institute of Biology Bucharest, Romanian Academy, 296 Splaiul Independenţei, Bucharest, Romania; 2 Terrestrial Ecology Research Group, Department of Ecology and Ecosystem Management, School of Life Science Weihenstephan, Technische Universität München, Freising, Germany; 3 Chair of Ecology and Biogeography, Nicolaus Copernicus University Torun, Toruń, Poland; University of Roehampton, UNITED KINGDOM

## Abstract

The theory of island biogeography predicts the effects of habitat isolation and size on species richness, community assembly, and the persistence of species. Various studies showed that habitat conditions and the ecology of species are also of relevance in explaining community assembly. Geographically isolated habitats like caves with rather constant environmental conditions provide models to test for the relevance of the above described variables. In this study we analysed springtails living in karst caves of the Romanian Carpathians and Dobrogea region. We considered phylogenetic relatedness, habitat and species characteristics to identify the relevant drivers of community assembly. Our data show that species richness of single caves is low. Neither phylogenetic relatedness nor habitat filtering and competitive interactions seem to shape species composition or to affect species richness. We found that glacial-interglacial cycles with subsequent range contractions and expansions might have led to independent and multiple colonisations of caves. Furthermore, single caves might have acted as refugia and thus might have provided the prerequisite for distinct evolution processes, leading to a high level of endemicity of these animal species.

## Introduction

The theory of island biogeography [1] and also its extensions onto multi-species metapopulation models [2] and neutral theory [3] explain island diversity by the trade-off between local colonisation and extinction, and as a function of island area and geographic isolation. A positive colonisation-extinction trade-off (the net colonization rate), in combination with large island size, increases richness [1]. Thus, the effective dispersal rate should be the major trigger of island species richness. Low dispersal rates do often not counteract local extinction events and thus reduce local and pool species richness in neutral [3, 4] and multi-species metapopulation models [2]. As a consequence of the competitive exclusion principle [5], strong competitive interactions might decrease local diversity even more [6].

However, dispersal also has influence on the spatial distribution of species and the spatial patterns of species occurrence, the geometry of occurrence. Extensive simulations of spatially explicit neutral models revealed that high dispersal is able to generate non-random patterns in this geometry [4, 7, 8]. Unfortunately, sampling data based on species lists, being most common in biogeographical studies, do not allow for the necessary parameterisation of neutral models, and thus do not allow for a direct test of model fit. However, neutral community assembly can be detected by the signatures of occurrence geometry. Ecological drift without dispersal limitation should cause a nested community assembly [9] where species poorer sites are true subsets of species richer sites [10]. Such a community assembly is equivalent to a passive sampling process where local sites are colonized from a regional species pool according to mass effects, as assumed by island biogeography [1]. Nested community organisation has gained particular interest among ecologists as it can be associated with community stability [11] and ecological gradients [9]. In turn, if colonization-extinction dynamics were of minor importance, which is the case for low dispersive cave relicts [12], the pattern of species co-occurrence should approach an equiprobable distribution, leading to comparably high species turnover between sites [4]. We note that this behaviour is a necessary but not a sufficient condition for nested community assembly. Other assembly processes, for instance environmental gradients or competitive interactions might also generate similar occurrence geometries.

In addition to the spatial pattern of species occurrences, low dispersal rates across regions and high levels of speciation within regions might also influence local phylogenetic community structures [13, 14]. Neutral community assembly randomizes the phylogenetic community structure. However, if competitive interactions might influence on local community compositions, Darwin’s [15] competition hypothesis predicts closely related species to have on average similar functional traits and co-occur less frequently than species being phylogenetically distant from each other. Consequently, local community structure should be phylogenetically overdispersed.

Habitat filtering, the selective process that picks potentially colonising species from the regional species pool according to environmental characteristics acts in the opposite direction. Filter processes should increase phylogenetic relatedness as species pass respective filters due to similar characteristics [16]. Thus, the analysis of phylogenetic community structure may serve as an additional element to assess the level of neutral community assembly. For instance, Niemiller and Zigler [17] reported increased taxonomic distinctness for cave-dwelling specialist species, cavernicoles, within individual caves in comparison to the regional species pool of cavernicoles; this indicates non-neutral effects of the local community assembly.

With respect to the low dispersive cavernicoles, island biogeography predicts low species richness in single caves in comparison to the regional species pool size. Consequently, β-diversity, the relationship between local (island) and regional richness measuring the variability in species composition between islands, should be high. Caves are insular habitats and often colonised by cavernicoles which are adapted to particular thermic, light, humidity, and resource conditions, and to the subterranean life [18, 19]. Many cavernicoles are cave-obligate (troglobionts) and have developed morphological and behavioural traits allowing them to dwell in dark and often moist environments [18].

Collembola have colonised nearly all terrestrial habitats, and are also prominent invertebrate cave-dwellers. Such troglobionts are known for several morphological and behavioural adaptations to the subterranean life. These include the reduction of eyes and pigmentation, the elongation of the extremities [20], as well as longer life span and higher resistance to starvation [18]. The entire set of convergent morphological features was termed troglomorphy by Christiansen [21] who showed that cavernicolous Collembola from different phylogenetic lineages share convergent features of claw shape and appendage lengthening.

Caves provide a natural laboratory to test hypotheses of island colonisation by organisms of generally low to very low dispersal ability [18]. Thus, springtails provide a useful model taxon for biogeographic studies in caves [22–24]. Here we analyse community structures of cave-dwelling springtails based on 141 cavernicolous Collembola species, recorded by repeated sampling of 189 karst caves of Romania. We complemented the occurrence data of taxa with cave environmental characteristics. Based on these data we address the following questions:

1. Is the local species richness depauperated and β-diversity increased with respect to the ecological drift models?
2. Does local community composition follow the neutral expectation?
3. Is the local phylogenetic community structure overdispersed as expected from competitive interactions?

## Material and methods

### Data set

We compiled 4,200 records of springtails collected from the karst caves of the Carpathian and Dobrogea region of Romania. These data were taken from 53 reliable literature sources (S1 Text). These data might include a few ‘tourist’ species that normally occur outside of caves. However, in nearly all cases it is unknown whether a certain species has occurred accidently or not. Importantly, invertebrates even temporarily associated with caves do not occur randomly among caves, but are associated to those with specific environmental features [25]. The number of sites with recorded species is a relative measure of sampling intensity prior used in analyses of subterranean fauna [26]. We assigned the caves into four classes according to the intensity of sampling: 1: 1–25% (less sampled / 1–3 number of visits); 2: 25–50% (moderately sampled / 4–6 number of visits); 3: 50–75% (highly sampled (7–9 number of visits); 4: 75–100% (very highly sampled / 10–12 number of visits). As the number of species generally increases with increasing intensity of data collection we excluded all caves which were surveyed fewer than three times. Based on these data we prepared a presence-absence matrix consisting of 141 springtail species assessed across 189 caves (S1 Table).

**Fig 1:**
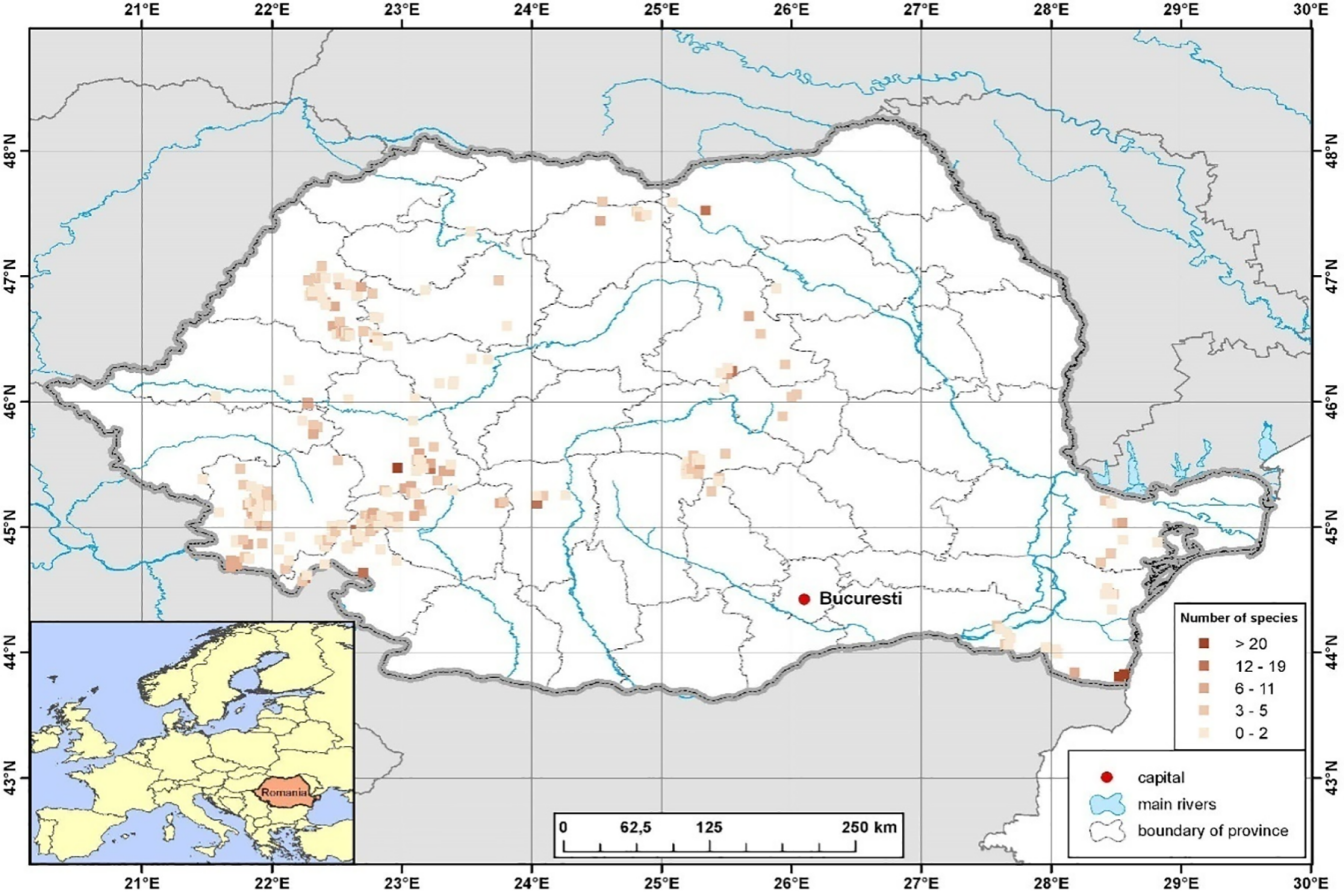
Study area with the exact geographic locations of all caves (N = 189) from which Collembola species assemblages were analysed.

For each cave we recorded its length, depth below ground, and the altitude of the entrance to characterize the subterranean habitat, and provide important predictors for subsequent analyses. Altitude is an approximate measure the relative richness of the surface fauna and thus the potential number of species that could colonize or become isolated in caves. Cave depth is a common proxi to habitat variety while the cave length conveys an important measure of the habitat size available [24]. These characteristics of each cave (geographic location, length, depth and altitude) were taken from Goran [27] and completed with data from the online data bank Speologie (http://www.speologie.org). The geographic location of all caves for which springtail species composition was analysed is displayed in Fig 1. All details on cave characteristics are given in (S2 Table).

### Phylogenetic analyses

We transformed memberships of nine taxonomic levels (species, subgenus, genus, tribe, subfamily, family, superfamily, suborder, order) into a dissimilarity matrix for all 141 species. We used the number of branchings from the lowest joint taxonomic unit to each species as the pair-wise dissimilarity [28, 29], thus *Anurida* (subgenus *Anurida) subarctica* (Poduromorpha, Neanuroidea, Neanuridae, Pseudachorutinae, Pseudachorutini) and the *Lepidocyrtus* (subgenus *Lanocyrtus*) *selvaticus* (Entomobryomorpha, Entomobryoidea, Lepidocyrtidae, Lepidocyrtinae, Lepidocyrtini) differ by 15 steps. From the taxonomic dissimilarity matrix we calculated for each cave the net relatedness index NRI that returns the standardised effect size (SES) of the mean phylogenetic distance [29]. To be in accordance with the common definition of SES we don’t use the negative complement here as Webb *et al*. [30] did. Consequently, NRI increases with decreasing phylogenetic clustering and reflects phylogenetic segregation (overdispersion) across the whole taxonomic tree.

### Statistics

We estimated the degree of β-diversity from proportional species turnover: β_P_ = 1-α/γ [31], where α is the average species richness per cave and γ the total number of cave-dwelling springtails in Romania. β_P_ quantifies the proportion of observed species that are not contained in a local community of average richness α. We assessed total species segregation among caves (negative species associations) by the C-score [32] that is a normalized count of the number of checkerboard submatrices ({{1,0},{0,1}} or {{0,1},{1,0}}). We performed a nestedness analysis to identify an ordered loss of species among caves [9] using the NODF (nestedness by overlap and decreasing fill) metric of Almeida-Neto *et al*. [33]. To obtain estimates on the variability of the C-score and NODF among the caves we used a shifting window approach and calculated both metrics for nine non-overlapping shifting windows each including 20 caves. Prior to analysis, caves were sorted according to longitude to include only nearby caves in each window.

In a pair-wise approach we calculated C-scores for each pair of species and used the respective standardised effect sizes (SES, see below) in major effects ANOVA with categorical species traits as predictors. As these came from both species of a pair we coded them as ‘1’ in case of trait identity and ‘0’ in case of trait difference. Most species do not interact ecologically leading to insignificant and randomly distributed SES scores. This makes a sound inference of effects challenging. Therefore, we adapted the approach of Lyons *et al*. [34] and pre-screened the data for significant (P < 0.05) positive and negative pairwise co-occurrences and used these species pairs only in the GLM analysis.

For statistical inference of NODF and the C-score, we used a null model approach and compared the observed co-occurrence metric scores with those obtained from 200 randomised matrices. As we wanted to infer the degree of ecological drift, we used two null models, one that accounted for unequal colonisation probabilities and one that did not. The proportional (PP) null model [35] resamples a matrix with placement probabilities proportional to observed row and column totals. Under the assumption that these reflect differences in cave (column totals) capacities and regional relative abundance (row totals) this null model should account for the ecological drift effect. Thus, this null model is equivalent to a neutral model without dispersal limitation and speciation. In turn the equiprobable (EE) null model resamples a matrix with linearly equiprobable placement probabilities and thus does not account for constraints of cave capacity and differential species colonisation probability. We calculated standardized effect sizes (SES = Obs–Exp) / StDev_Exp_; Obs and Exp: observed and expected scores, StDev_Exp_: standard deviation of expectation). SES scores should have values below –1.96 and above +1.96 at the two-sided 5% error level under the assumption that the respective null distribution is approximately normal. We used these SES values in main effects general linear models to link species functional traits, cave characteristics, and phylogenetic relatedness with the pattern of species co-occurrences. To account for possible spatial non-independence of caves we included the dominant eigenvector of the Euclidean distance matrix of caves into the regression model. Errors refer to standard errors. Co-occurrence and null model calculations were done with the freely available Fortran software Turnover (available from WU by request). Regression analysis and ANOVA was based on the general linear model module of Statistica 12.

## Results

In total, we recorded 141 cavernicolous springtail species across our study region (S1 Table). The species richness per cave was comparatively low with a median of 4 species, ranging from one to 30 species. In consequence, ß-diversity was very high (ß = 0.96±0.01). Only one species, *Lepidocyrtus serbicus*, occurred in more than half of the caves (S1 Table). In turn, 37 species (23.7%) of all species were endemic to one single cave.

**Fig 2:**
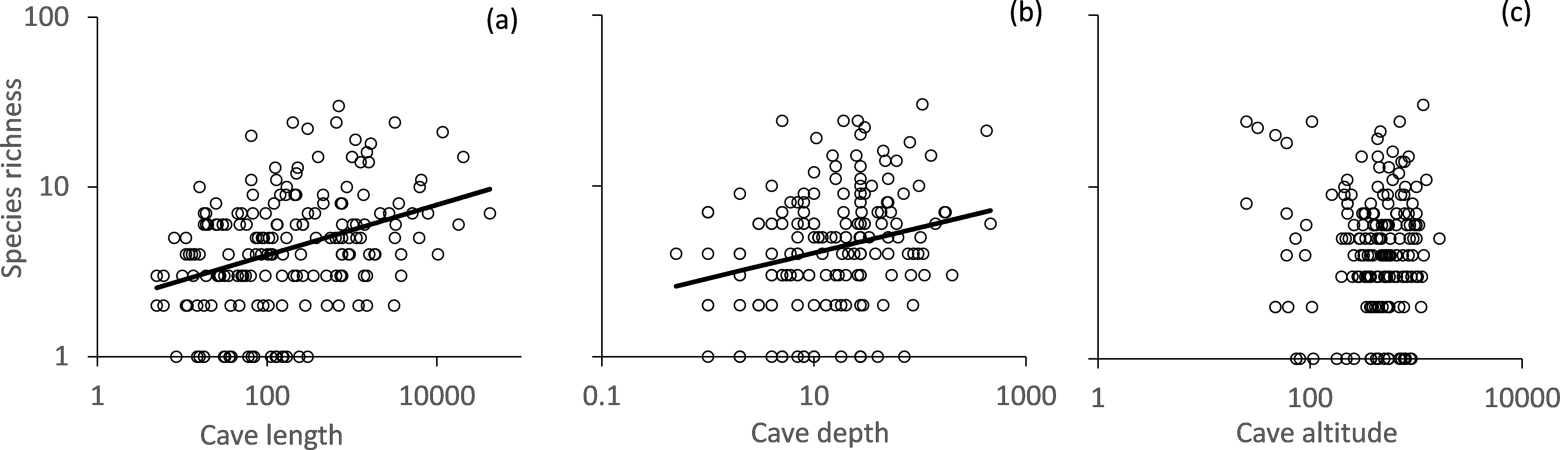
Local species richness of Romanian cave dwelling springtails increased significantly with cave length (permutation r^2^ = 0.13, P < 0.001), cave depth (r^2^ = 0.06, P < 0.01), but not with cave altitude (P < 0.1). Data from 189 caves.

Local springtail richness increased allometrically with cave length (Fig 2A) and cave depth (Fig 2B), while altitude showed no significant effect on springtail richness (Fig 2C). Subsequent linear modelling (Table 1) retained cave length as the most important variable explaining richness.

**Table 1:** General linear modelling (given are partial η^2^ values) indicated cave length to be positively (indicated by the + sign) associated with local cave-dwelling springtail species richness (α-diversity). The dominant eigenvector of the cave Euclidean distance matrix (EV1) served as covariate to account for possible spatial non-independence of richness and phylogenetic relatedness. The net relatedness index increased with richness and decreased with geographical distance of caves. N = 189.

Variable	a-diversity	NRI
Altitude	<0.01	<0.01
Cave length	(+) 0.07***	<0.01
Cave depth	<0.01	<0.01
Species richness	-	(+) 0.04**
EV1	<0.01	(-) 0.03**

**: P(F) < 0.01

*** P(F) < 0.001

**Fig 3:**
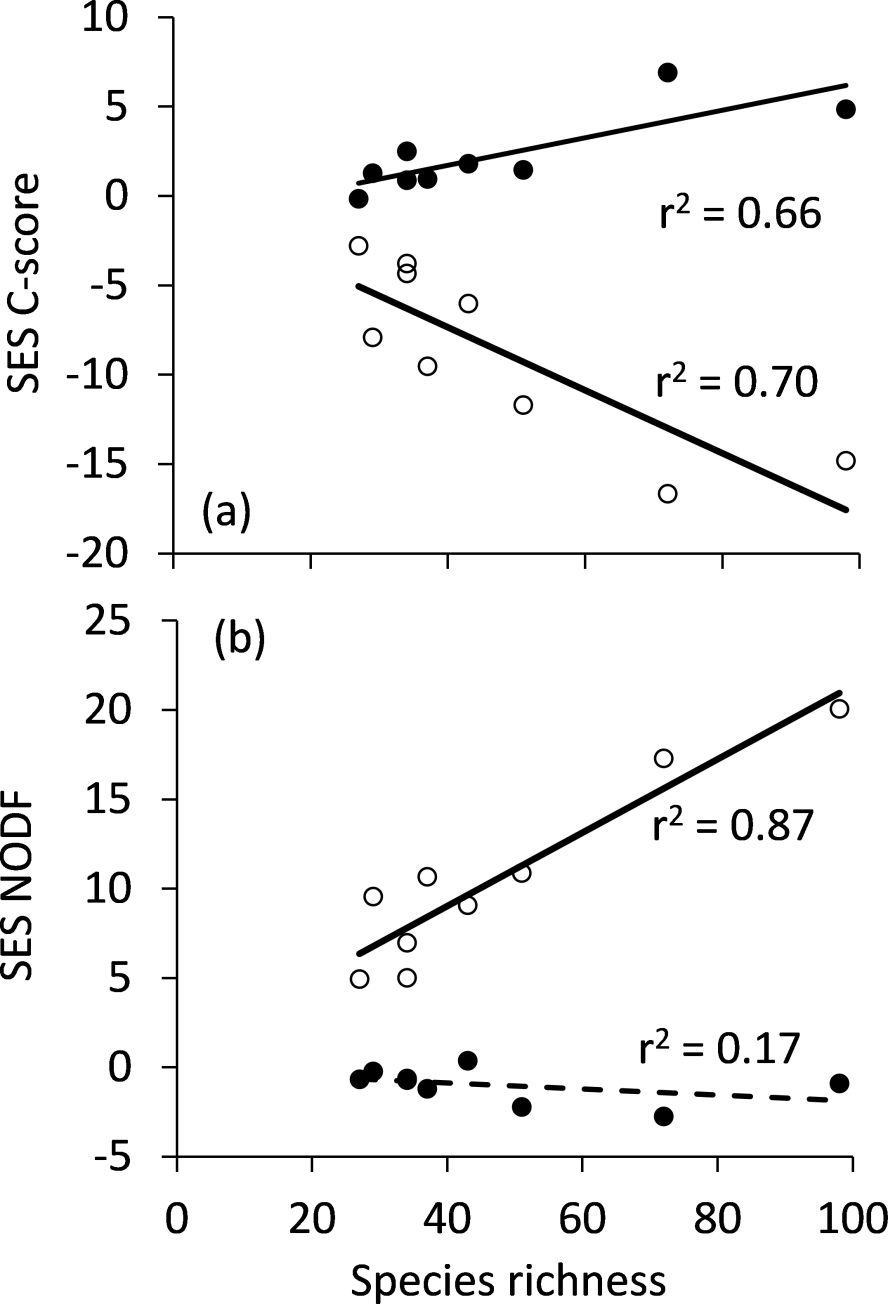
The degree of species segregation (C-score, a) and nestedness (NODF, b) quantified by the standardised effect sizes SES of the equiprobable (open dots) and the fixed–fixed (black dots) null models in dependence on species richness in nine shifting windows of 20 caves each. Full linear OLS regressions a) and b): permutation P < 0.01, Broken regression line in b): permutation P > 0.10.

Species co-occurrences were not segregated (Fig 3A) and highly nested (Fig 3B) when compared to the equiprobable (EE) null model. The degree of nestedness increased and the degree of spatial segregation decreased with increasing species richness per shifting window (Fig 3). In turn, according to the proportional (PP) null model species co-occurrences appeared to be significantly segregated (Fig 3A) and not significantly nested (Fig 3B). The pattern of species co-occurrence was not significantly linked to cave characteristics (length, depth, altitude) (Table 1).

**Fig 4:**
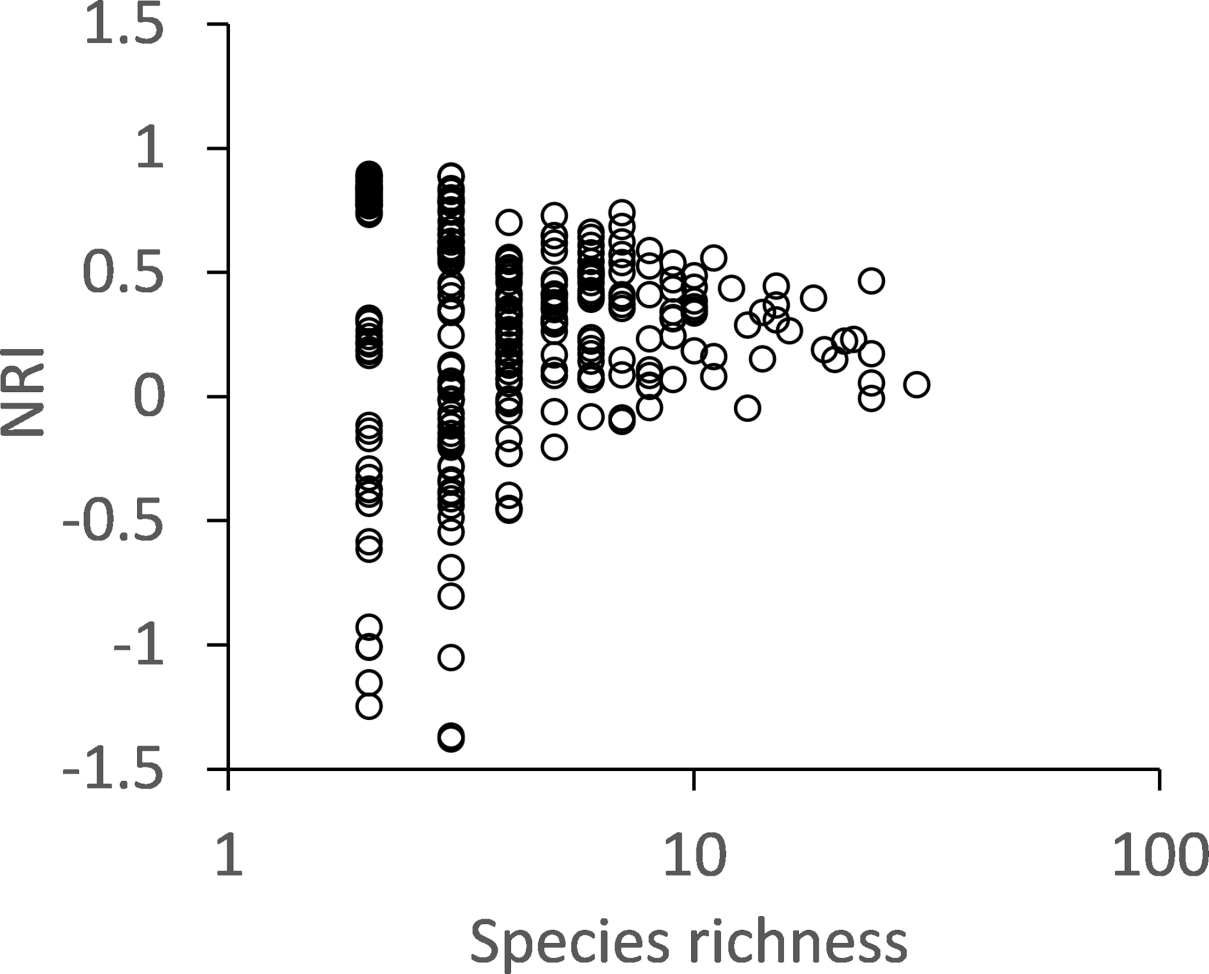
The net relatedness index (NRI) of local springtail assemblages was weakly positively related to cave species richness (P < 0.01; Table 1) but did not deviate from random expectation (equiprobable null model) that expects NR values < |2.0| at the two-sided 5% error level.

Phylogenetic community structure did not deviate from the equiprobable null standard (Fig 4) and was not related to cave characteristics (Table 1). NRI weakly increased with species richness (Table 1, Fig 4).

## Discussion

In line with our first research question, we assumed that local springtail cave diversity is comparably depauperated. We found clear indication in favour of this hypothesis. An average of only four species per cave in combination with the maximum of 30 species found in one cave is very low compared to typical species numbers in European ecosystems, where even in single soil samples frequently more than ten springtail species occur, and temperate forests harbour well above 50 species [36] and grasslands below 40 species [37]. Importantly, the present analysis is based on all species found in the respective caves. With a single exception [20] the sources used do not differentiate between the species richer cave entries were facultative troglobionts may be found and the more depauperate middle parts of the caves. Future work needs to study spatial patterns of obligate terrestrial cave-dwelling springtails at larger scale.

Under ecological drift such low levels of species richness might be due to very restricted dispersal. Strong interspecific competition might also cause local extinction. However, cavernicole springtails exhibit a wide range of morphological and functional characters indicating sufficient niche differentiation to minimize competitive exclusion. Additionally most caves provide sufficient habitat heterogeneity to allow local co-existence [38, 39]. However, total springtail richness is apparently limited by the limited food availability particularly in deeper parts of caves [17, 40, 41]

As the proportional turnover β_P_ ranges from zero (regional faunal homogeneity) to 1.0 (perfect local faunal uniqueness), the observed β-diversity of 0.96 implies a nearly perfect turnover in faunal composition between caves. Each cave shows a distinct springtail community. In turn, cavernicole springtails (156 species) account for 37% of the total Romanian fauna (434 species, Fiera, pers. comm.). In this respect we note that regional allometric species—sample size relationships of animal and plants (S_regional_ = S_local_N^z^) rarely have slopes above z = 0.30 [42]. Such values occur in small local samples and among isolated habitats and islands. In comparison, our data show that the Romanian cave springtails have an extraordinary high slope of 0.70, that is very rarely found among terrestrial taxa [42, 43]. Therefore, under ecological drift without dispersal limitation we might expect an average local richness of at least 32 species among the 189 caves.

In isolated habitat patches and on islands, species richness generally decreases with increasing altitude, and increases with habitat size and heterogeneity [43]. Cave springtails [44] as well as other cavernicoles [45, 46] do not clearly follow this richness pattern. In our study, species richness was only weakly correlated with cave length, depth, and altitude (Table 1, Fig 2). Importantly, cave length, as a measure of habitat size [47], explained only 13% of total variance in species richness (Fig 2). Again this finding is best explained by cave isolation.

Only few previous studies reported on springtail richness patterns in caves. Dányi [48] reported 67 species from 22 caves in Hungary. Barjadze et al. [49] identified 47 species from 56 caves in Georgia (another 18 were determined at genus level). 67 species could be detected in caves in Germany [50]. Christian and Spötl [51] found 26 species across Austria. Thus, the Romanian cavernicole springtail diversity seems to be extraordinary high. As we have counted only obligatory and facultative cavernicoles, these differences are not due to the inclusion of ‘tourist’ species sometimes recorded from the entrances of caves. This might be due to the fact that Romania provides many isolated karst caves that were colonized after subsequent glacial stages followed by distinct speciation processes. Glacial-interglacial cycles are known as an important motor for species diversification across Europe and North America [52], and were shown for various invertebrates at the interspecific and intraspecific level [53], as well as for cavernicoles at taxon level [54]. These severe climatic changes caused range extractions and range expansions of most species [53]. Thus, caves might have been colonised multiple times and independently from each other. Furthermore, current studies showed that caves acted as important refugia where many species persisted during glacial stages, so that many springtail taxa found in distinct caves are relicts of the glacial period, being endemic to one single cave [20]. Importantly, both processes, colonization and persistence, are not mutually exclusive and rapid speciation in relict species seems to be common [20].

For the eastern USA, Christman et al. [55] and Niemiller and Zigler [17] also reported spatially restricted distributions of springtails in caves. Many species were shown to be endemic to only a single cave. Similar to our findings, the latter study also found an average of only three species per cave. This finding is a strong indication that richness patterns are not governed by habitat properties or climate regimes as these differ widely between the Romanian and the US caves. Richness patterns were also not influenced by morphological and ecological characteristics as these did not significantly influence patterns of occurrence (Table 1, Fig 4).

Our second starting question anticipated that spatial patterns in species co-occurrences might indicate deviations from neutral community assembly. Indeed the pattern of species co-occurrence was shifted towards a nested distribution (Fig 3B) and did not significantly differ from a passive sampling pattern. In line with the extraordinary high species turnover between caves, the pattern of co-occurrence was significantly segregated according to both standards, the equiprobable and proportional null model expectations (Fig 3A). Both finding are again best explained by neutral cave colonization with very low dispersal.

Finally we assumed that phylogenetic community structure should reflect species interactions and possible filter effects. This was not the case (Table 1). Phylogeny, as quantified by the net relatedness index NRI, was only weakly related to cave richness, possibly due to statistical biases at low richness. In none of the caves did NRI deviate from equiprobable random expectation (Fig 4). This lack of pattern deviates from most terrestrial habitats [56], where community structure exhibits at least moderate phylogenetic signals due to filter effects, and species of similar ecological requirements and characters pass habitat filters [57]. Interspecific competition is also able to induce phylogenetic signals in cases where species with similar characters exclude each other [58]. These species are on average more closely related than expected from a random sample from the associated species pool, in our case possible cave dwellers. In turn, lack of phylogenetic signal is expected under neutral community assembly.

In conclusion, our study suggests that communities of cave dwelling springtails have mainly been formed by postglacial colonization-extinction dynamics, combined with extremely low dispersal processes. Apparently, habitat filters and competitive interactions did not markedly affect community composition and local species richness.

## Supporting information

S1 Text References used for compiling species distribution in caves. (DOCX)

S1 Table Presence-absence matrix of Collembola in Romanian caves. (XLSX)

S2 Table Cave characteristics. (XLSX)
