# Supplementary material for: Neutral colonisations drive high beta-diversity in cavernicole springtails (Collembola)
Source: PLoS One. 2018 Jan 2;13(1):e0189638. doi: 10.1371/journal.pone.0189638 (PMC5749714; doi:10.1371/journal.pone.0189638)
Supplement: S1 Text — (DOCX) [file pone.0189638.s001.docx]

**Neutral colonisations drive high beta-diversity in cavernicole springtails (Collembola)**

Cristina Fiera, Jan Christian Habel, Werner Ulrich

**References used for compiling occurrences of Collembola in Romanian caves**

1. Boitan, V., & Negrea, S. (2001). Contribution to the knowledge of the terrestrial cavernicolous fauna from the middle Cerna Valley (Banat, Romania). Travaux du Muséum National d'Histoire Naturelle “Grigore Antipa”*, 33,* 1–22.
2. Botoşăneanu, L. (1971). Observation sur la faune aquatique hypogée des Monts du Banat (Roumanie). *Trav. Inst. Sp*é*ol.* “*Emil Racovitza*”*,* *10,* 123–166.
3. Burghele-Bălăcescu, A., & Avram, Ş. (1966). Peşteri cercetate în Oltenia între Valea Motrului şi Valea Tismanei. *Lucrările Institutului de Speologie* “*Emil Racoviţă*”*, 5,* 21–41.
4. Dancău, D., & Tăbăcaru, I. (1964). Observaţii zoogeografice asupra faunei cavernicole din Oltenia şi Banat. *Lucrările Institutului de Speologie* “*Emil Racoviţă*”*, 3,* 293–340.
5. Decu, V. G., Negrea, A., & Negrea, Ş. (1974). Une oasis biospéologique tropicale développée dand une région tempérée: Peştera lui Adam de Băile Herculane (Carpates méridionales, Roumanie). *Trav. Inst. Sp*é*ol.* “*Emil Racovitza*”*,* *13,* 81–103.
6. Dumitrescu, M., & Orghidan, T. (1969). Date noi obţinute în studiul faunei litoclazice. *Lucrările Institutului de Speologie* “*Emil Racovitza*”*,* *8,* 55–71.
7. Dumitrescu, M., Tanasachi, J., & Orghidan, T. (1955). Contribuţii la studiul biologiei chiropterelor. Dinamica şi hibernaţia chiropterelor din pestera liliecilor de la Mănăstirea Bistriţa. *Buletin ştiintific, Secţiunea de ştiinţe biologice, agronomice, geologice şi geografie,* *7(2),* 317–357.
8. Dumitrescu, M., Orgidan, T., Tanasachi, J., & Georgescu, M. (1965). Contribuţii la studiul monografic al Peşterii de la Limanu. *Lucrările Institutului de Speologie* “*Emil Racovitza*”*,* *4*, 21–58.
9. Dumitrescu, M., Orgidan, N., Orghidan, T., Puşcariu, V., Tanasachi, J., Georgescu, M., & Avram, Ş. (1967). Contribuţii la studiul peşterilor ȋn regiunea Hunedoara. *Lucrările Institutului de Speologie* “*Emil Racovitza*”*,* *6,* 9–88.
10. Gisin, H., & Gama, M.M. da (1971). Notes taxonomiques et evolutives sur trois especes nouvelles Pseudosinella cavernicoles provenant de Yougoslavie et de Roumanie. *Revue Suisse de Zoologie,* *78,* 217–225.
11. Gruia, M., & Ilie, V. (2000-2001). Collembola of the karstic system of Romania (II). *Trav. Inst. Sp*é*ol.* “*Emil Racovitza*”*,* *40,* 63–122.
12. Gruia, M., & Popa, I. (2004-2005). Collembola of Piatra Craiului National Park (II). *Travaux de l'institut de Sp*é*ologie* “*Émil Racovitza*”*,,* *43-44,* 119–131.
13. Gruia, M. (1964). Contribuţii la studiul collembolelor din România. *Studii şi cercetări de biologie. Seria Zoologie, 16(3),* 197–204.
14. Gruia, M. (1965a). Contribuţii la studiul collembolelor din România. *Travaux de l’Institut de Spéologie* “*Émile Racovitză*”*, 4,* 191–202.
15. Gruia, M. (1965b). Specii noi de collembole din peşterile României. *Studii şi cercetări de biologie, Seria Zoologie,* *17(2),* 149–155.
16. Gruia, M. (1966a). Collembole noi pentru fauna României. *Studii şi cercetări de biologie, Seria Zoologie,* *18(3),* 247–253.
17. Gruia, M. (1966b). Specii noi de Onychiuridae şi Entomobryidae pentru fauna României. *Travaux de l’Institut de Spéologie* “*Émile Racovitză*”*, 5*, 185–194.
18. Gruia, M. (1967a). Sur quelques Collemboles cavernicoles de Roumanie. *International Journal of Speology,* *3,* 97–109.
19. Gruia, M. (1967b). Trei specii de Hypogastruridae din fauna de colembole a României. *Travaux de l’Institut de Spéologie* “*Émile Racovitză*”*,* *6,* 173–181.
20. Gruia, M. (1967c). Collemboles provenant du milieu lapidicole et lithoclassigule. Revue d'écologie et de biologie du sol*,* *4(2),* 313–322.
21. Gruia, M. (1969a). Date asupra răspândirii colembolelor în peşterile României. *Travaux de l’Institut de Spéologie* “*Émile Racovitză*”*,* *8,* 161–178.
22. Gruia, M. (1969b). *Xenylla tanasachiae* n. sp. – une nouvelle Collembole cavernicole de Roumanie. *Reichenbachia,* *12(2),* 15–19.
23. Gruia, M. (1970). Considerations sur deux especes aveugles de Collemboles cavernicoles de Roumanie: *Acherontides spelaea* (Ionesco) et *A. tanasachiae* (Gruia). *Trav. Inst. Spéol,* “*Émile Racovitză*”*,* *9,* 197–199.
24. Gruia, M. (1971). Sur certaines espèces d’Onychiuridae (Collembola) des grottes de Roumanie. *Acta Zoologica Cracoviensia,* *16(4),* 283–288.
25. Gruia, M. (1972). Quelques espèces nouvelles de Collemboles cavernicoles. *Travaux de l’Institut de Spéologie* “*Émile Racovitză*”*,* *11,* 257–264.
26. Gruia, M. (1973). *Onychiurus (Oligaphorura) multiperforatus*, nouvelle espèce d’ Onychiuridae de Romanie. *Travaux de l’Institut de Spéologie* “*Émile Racovitză*”*,* *12*, 173–176.
27. Gruia, M. (1974). Deux collemboles (Entomobryinae) nouveaux de la faune cavernicole de Roumanie. *Travaux de l’Institut de Spéologie* “*Émile Racovitză*”*,* *13,* 55–59.
28. Gruia, M. (1975). Collembole subterane din România. *Institutul de Ştiinţe Biologice, Bucureşti*, unpublished Phd thessis. 197 pp.
29. Gruia, M. (1989). Nouvelles especes troglobiontes des Collemboles de Roumanie. *Misc. speol. Rom., 1,* 103–111.
30. Gruia, M. (1994). Les Oncopodura (Collembola, Oncopoduridae) des Grottes de Roumanie. *Mémoires de Biospéologie,* *21*, 67–74.
31. Gruia, M. (1996). Quelques considerations sur la faune de Collemboles de la grotte de Movile, Roumanie*. Mémoires de Biospéologie,* *23*, 105–109.
32. Gruia, M. (1998a). Collembola of the karstic system from Movile. *Travaux de l’Institut de Spéologie* “*Émile Racovitză*”*,* *37-38,* 167–174.
33. Gruia, M. (1998b). Sur la faune de Collemboles de l’écosystème exokarstique et karstique de Movilé (Dobrogea du sud, Mangalia, România). *Mémoires de Biospéologie* *25,* 45–52.
34. Gruia, M. (1999). Concerning some Onychiuridae species (Apterygota, Collembola) from the shafts in the Retezat Mountains (Romania), alpine areas. *Mémoires de Biospéologie,* *26,* 11–16.
35. Gruia, M. (2003). Collembola from Romanian caves. Travaux du Muséum National d'Histoire Naturelle “Grigore Antipa”*, 35,* 139–158.
36. Ionescu, C.N. (1914). Contributions à la faune des insectes Collemboles de Roumanie (en comprenant aussi des formes cavernicoles). *Bulletin de la Section Scientifique de l’Academie Roumane,* *7,* 220–225.
37. Ionescu, C.N. (1915). Contributions à la faune des insectes Collemboles (terrestres, cavernicoles et aquatiques) de Roumanie. *Annales Scientifiques de l’Université de Jassy*, *9(3–4),* 463–518.
38. Ionescu, C.N. (1922). Quelques nouveaux insectes Collemboles recoltes dans les grottes des Carpathes meridionales. *Annales Scientifiques de l’Université de Jassy,* *11(3–4),* 373–382.
39. Ionescu, M.A. (1951). Contribuţii la studiul Collembolelor (Insecta: Apterygota) din România. *Buletin Ştiinţific, Secţiunea de Ştiinţe Biologice, Agronomice, Geologice şi Geografice,* *3(4),* 639–651.
40. Mack-Firă, V. (1961). Contribuţii la studiul Onychiuridelor (Collembola) din România. *Analele Universităţii C. I. Parhon*, *Seria Ştiinţele Naturii,* *18,* 191–196.
41. Nae, A., Vlaicu, M., Popa, I., Constantinescu, T., Iavorschi, V., & Niţu, E. (2004-2005). First note on the invertebrate fauna of caves in the Piatra Craiului National Park. *Travaux de l’Institut de Spéologie* “*Émile Racovitză*”*,* *43*, 133–164.
42. Negrea, A., Negrea, Ş., & Karban, G. (1994). Grottes explorees dans le basin du Doman (Banat, Roumanie). *Trav. Inst. Speol.* “*Émile Racovitză*”*,* *33,* 179–194.
43. Negrea, Ş., & Negrea, A. (1972). Recherches sur l'association pariétale des grottes du Banat (Romanie). *Acta zool. cracov., 35(5),* 45–95.
44. Negrea, Ş., & Negrea, A. (1979). Peşterile din defileul Dunării şi fauna terestră. In T. Orghidan & Ş. Negrea (Eds.), Speologia (pp. 30–75). Bucureşti: Editura Academiei, RSR.
45. Negrea, Ş., & Negrea, A. (1983). Considerations sur les ecosystemes des grottes Comarnic et Popovăţ (Banat, Roumanie). *Trav. Inst. Speol.* “*Émile Racovitză*”*,* *22,* 47–55.
46. Negrea, Ş., & Negrea, A. (1971). Sur la synusie du guano des grottes du Banat (Roumanie). *Trav. Inst. Speol.* “*Émile Racovitză*”*, 10,* 81–122.
47. Negrea, Ş., & Negrea, A. (1977). Sur les associations de plancher des grottes du Banat (Roumanie). *Trav. Inst. Speol.* “*Émile Racovitză*”*,* *16,* 99–139.
48. Negrea, Ş., & Negrea, A. (1969). Peşterile din defileul Dunării. *Lucr.* *Trav. Inst. Spéol.* “*Émile Racovitză*”*,* *8*, 25–50.
49. Nitzu, E., Nae, A., Popa, I. (2006-2007). Eco-faunistic study on the invertebrate fauna (Araneae, Collembola and Coleoptera) from the Vârghiş gorge natural reserve (Eastern Carpathians, Romania), with special note on the micro- refugial role of the subterranean habitats. *Trav. Inst. Spéol.* “*Émile Racovitză*”*,* *45-46,* 31–50.
50. Nitzu, E., Popa, I., Nae, A., & Iusan, C. (2008). Faunal researches on the invertebrates (Coleoptera, Orthoptera, Collembola and Araneae) in the Rodnei Mountains Biosphere Reserve. *Trav. Inst. Spéol.* “*Émile Racovitză*”*,* *47,* 3–52.
51. Pleşa, C., Moldovan, O., & Munteanu, A. (1996). Aperçu biospéologique sur la grote de Vadu-Crişului, Monts Pădurea Craiului (Transylvanie, Roumanie). *Trav. Inst. Spéol.* “*Émile Racovitză*”*,* *35,* 115–142.
52. Popa, I., & Gruia, M. (2006). Research in the Piatra Craiului National Park. In O. G. Pop & Hanganu H. (Eds*.*), Collembola of the Piatra Craiului National Park, Vol. 3, (pp. 172–176). Braşov: Editura Universităţii Transilvania.
53. Viehmann, I., Pleşa, C., & Rusu, T. (1964). Peştera de la Vadu-Crişului. *Lucrările Institutului de Speologie "Emil Racoviţă," 3*, 49–81.
